# Crouzon syndrome: preimplantation genetic testing for a familial case with a whole and a mosaic variant of the disease

**DOI:** 10.18699/vjgb-25-75

**Published:** 2025-09

**Authors:** E.V. Soloveva, M.M. Skleimova, L.I. Minaycheva, A.F. Garaeva, E.M. Bakulina, E.A. Ladygina, O.R. Kanbekova, G.N. Seitova

**Affiliations:** Research Institute of Medical Genetics, Tomsk National Research Medical Center of the Russian Academy of Sciences, Tomsk, Russia; Research Institute of Medical Genetics, Tomsk National Research Medical Center of the Russian Academy of Sciences, Tomsk, Russia; Research Institute of Medical Genetics, Tomsk National Research Medical Center of the Russian Academy of Sciences, Tomsk, Russia; Research Institute of Medical Genetics, Tomsk National Research Medical Center of the Russian Academy of Sciences, Tomsk, Russia; Vitromed LLC, Novosibirsk, Russia; Vitromed LLC, Novosibirsk, Russia; Regional Perinatal Center named after I.D. Yevtushenko, Tomsk, Russia; Research Institute of Medical Genetics, Tomsk National Research Medical Center of the Russian Academy of Sciences, Tomsk, Russia

**Keywords:** Crouzon syndrome, PGT-M, preimplantation genetic testing, IVF, mosaicism, FGFR2 gene, синдром Крузона, ПГТ-М, преимплантационное генетическое тестирование, ЭКО, мозаицизм, ген FGFR2

## Abstract

Crouzon syndrome, which is a hereditary craniosynostosis, can be the result of inheritance from either parent, as well as de novo mutations in the FGFR2 gene. With a confirmed molecular genetic diagnosis, preimplantation genetic testing for monogenic diseases (PGT-M) is available for high-risk families. However, there is currently little information in the literature about using this approach to prevent this condition. The aim of our study was to describe the clinical case of IVF/ICSI with PGT-M for Crouzon syndrome with a successful outcome and confirmatory diagnostics. PGT-M was planned and performed for a married couple (aged 24 and 25), in which the husband had Crouzon syndrome. The husband’s father had a milder form of Crouzon syndrome and the pathogenic variant of the FGFR2 gene was in a mosaic form. During preparation, a testing system was selected for the pathogenic variant NM_000141.5(FGFR2):c.1007A>G (p.Asp336Gly) of the FGFR2 gene, and gene-linked polymorphic microsatellite markers. The STR markers in the husband’s father excluded chimerism for the pathogenic variant and indicated mosaicism with the involvement of germ cells. Molecular genetic analysis was performed using а nested PCR, with detection by fragment analysis for STRs and restriction analysis of the pathogenic variant. During the IVF program, superovulation stimulation and embryological procedures were performed according to standard protocols. Fertilization was achieved using the ICSI method, and blastocyst biopsy was done on the sixth day of development. For PGT-M, a direct analysis of pathogenic variants and an indirect analysis of selected informative STRs were used. The thawed embryos were transferred based on the results of preimplantation testing. We selected twelve STRs flanking the FGFR2 gene, eight informative ones were used during PGT-M. In the IVF program, 15 mature oocytes were obtained, then four blastocysts were biopsied. One of the four embryos inherited a normal paternal chromosome, the other three had the pathogenic variant and the associated risk haplotype. A singleton pregnancy has occurred as a result of embryo transfer recommended after PGT-M. Following the child’s birth, molecular diagnostics were performed, confirming the PGT-M result. The presented clinical case provides an effective example of IVF with PGT-M to prevent the birth of affected children in families with hereditary craniosynostosis.

## Abstract (ru)

Синдром Крузона, относящийся к наследственным краниосиностозам, может быть как результатом наследования от одного из родителей, так и вариантом de novo мутаций в гене FGFR2. При подтвержденном молекулярно-генетическом диагнозе для семей высокого риска возможно проведение преимплантационного генетического тестирования моногенного заболевания (ПГТ-М). Однако на сегодняшний день в литературе мало сведений о таком подходе к профилактике данного заболевания. Целью нашей работы явилось описание клинического случая ЭКО/ ИКСИ с ПГТ-М для синдрома Крузона с успешным исходом и подтверждающей диагностикой. Планирование и проведение ПГТ-М выполнены для супружеской пары (24 и 25 лет), в которой синдром Крузона был у мужа. У отца супруга синдром Крузона был в более легкой форме и патогенный вариант гена FGFR2 присутствовал в мозаичном варианте. На подготовительном этапе подобрана система тестирования патогенного варианта NM_000141.5(FGFR2):c.1007A>G (p.Asp336Gly) гена FGFR2 и сцепленных с геном полиморфных микросателлитных маркеров. Профиль STR-маркеров у отца супруга исключал химеризм по патогенному варианту и свидетельствовал о мозаицизме, затрагивающем половую линию. Молекулярно-генетический анализ проводили методом гнездовой ПЦР с детекцией фрагментным анализом для STR и рестрикционного анализа для патогенного варианта. В ходе программы ЭКО стимуляцию суперовуляции и эмбриологические процедуры выполняли по стандартным протоколам, оплодотворение проводили методом ИКСИ. Биопсия бластоцист выполнялась на 6-е сутки развития. При ПГТ-М использовали прямой анализ патогенного варианта и косвенный анализ по отобранным на подготовительном этапе информативным STR. Перенос размороженного эмбриона выполнен с учетом результатов преимплантационного тестирования. Нами было отобрано двенадцать STR, фланкирующих ген FGFR2, из них восемь информативных были использованы в ходе ПГТ-М. В программе ЭКО получено 15 зрелых ооцитов и в дальнейшем четыре бластоцисты, которые были биопсированы. Один из четырех эмбрионов унаследовал нормальную отцовскую хромосому, в трех остальных выявлялся патогенный вариант и ассоциированный с ним гаплотип риска. В результате переноса в полость матки эмбриона, рекомендованного по результатам ПГТ-М, наступила одноплодная беременность. После рождения ребенка проведена молекулярно-генетическая диагностика, подтвердившая результат ПГТ-М. Представленный нами клинический случай демонстрирует эффективный пример применения ЭКО с ПГТ-М для предотвращения рождения больных детей в семьях с наследственными краниосиностозами.

## Introduction

Crouzon syndrome belongs to an extensive heterogeneous group of craniosynostosis – birth defects that are characterized by premature fusion of one or more sutures in the cranial vault prior to the completion of growth and development of the brain, which leads to limited growth of the skull, brain, face and central nervous system development. Among craniosynostosis, syndromic craniosynostoses are estimated to comprise 15 % of all cases. To date, there are over 180 craniosynostosis syndromes identified. About 8 % of cases are familial or inherited (Al-Namnam et al., 2019). Crouzon syndrome (OMIM 123500) is the most common syndrome among hereditary craniosynostosis. It is caused by mutations in the fibroblast growth factor receptor 2 (FGFR2) gene, which is located on chromosome 10 (10q26.13). The FGFR2 protein is involved in cell signaling, and disruption of the FGFs/FGFR2 signaling pathway leads to abnormal differentiation, proliferation and apoptosis (Al-Namnam et al., 2019; Yapijakis et al., 2023).

The incidence of Crouzon syndrome is approximately 16.5 cases per million live births (1:60,000). We have not found any data on the incidence of the disease in Russia. The disease is inherited as an autosomal dominant trait with incomplete penetrance and variable expressivity. In approximately 70 % of cases the disease is inherited from one parent, while in other cases, it is the result of a de novo mutation (Al-Namnam et al., 2019). The syndrome was first described by Louis Edouard Octave Crouzon in 1912.

The disease usually manifests itself in the first three years of life. It can be suspected during the antenatal period using ultrasound examination. It is also often detected at birth due to its classic signs in newborns, which include craniosynostosis, hypoplasia of the middle part of the face, proptosis (exophthalmos), and, in some cases, a beaked-shaped nose. Other common manifestations of the syndrome include coronary craniosynostosis with other cranial sutures fusion, brachycephaly, hypertelorism, prominent frontal tubercles, strabismus, orbital proptosis, mandibular prognathism, and maxillary hypoplasia. These signs either become more pronounced or may regress over time (Al-Namnam et al., 2019). Hearing loss is also common (55 %), and fusion of the C2 and C3 vertebrae occurs in 30 % of cases. Another manifestation may be progressive hydrocephalus (30 %). The patients’ mental abilities are usually normal, but in some cases, increased intracranial pressure can lead to intellectual disability. The differential diagnosis of Crouzon syndrome includes Apert syndrome and Pfeiffer syndrome. Historically, these diseases were described separately, but the overlap of spectrum of molecular genetic disorders suggests that these conditions form a continuum (Koltunov, 2011; Al-Namnam et al., 2019).

Treatment of Crouzon syndrome is based on the severity of symptoms. To optimize treatment, a comprehensive assessment by a multidisciplinary team of specialists is needed. The main treatment method is surgery. It allows to perform correction of the face skull and eye sockets in order to optimize cerebral blood flow and prevent the effects of increased intracranial pressure, blindness and mental retardation. Surgical intervention can be staged or combined, depending on the level of functional impairment in the patients and their age (Koltunov, 2011; Sokolova et al., 2024).

Molecular genetic examination is currently an important part in the diagnosis of Crouzon syndrome. If there is a high risk of transmission of Crouzon syndrome to offspring, preimplantation genetic testing for monogenic disease (PGT-M) may be used (Wenger et al., 1993–2025). To achieve this, the couple needs to resort to IVF (in vitro fertilization). PGT-M is currently used worldwide for a wide range of monogenic diseases, however, we found in the literature a description of only one clinical case of PGT-M for Crouzon syndrome (Abou-Sleiman et al., 2002).

In our work, we presented a clinical case of IVF/ICSI with PGT-M with a successful outcome and confirmatory diagnostics.

## Materials and methods

IVF with PGT-M was performed for a married couple (a 24-year-old woman and a 25-year-old man), in which the husband had the disease status of Crouzon syndrome. The phenotypic manifestations of the man included hypertelorism, a beaked nose, exophthalmos (not very pronounced), hypoplasia of the middle part of the face, a high palate and a protruding chin. His father also had Crouzon syndrome. The father’s clinical manifestations were milder, with no exopthalmos, hypoplasia, or hypertelorisms. Familial pathogenic variant of NM_000141.5(FGFR2):c.1007A>G (p.Asp336Gly) was detected in a heterozygous state in the spouse and his father as a result of Sanger sequencing; “14 % mosaicism or chimerism with heterozygosity” for the father was noted (Genoanalytika LLC, Moscow, 2022). The abbreviation for the pathogenic variant, D336G, is used in this article. The variant is described in the ClinVar database (Variation ID: 374815) and is associated with the development of Crouzon syndrome (OMIM 123500). At least three in silico pathogenicity prediction programs confirmed the pathogenic effect of the variant on the gene or gene product (AlphaMissense, Revel, Aggregated Prediction). The nucleotide sequence variant was not registered in the control sample of The Genome Aggregation Database v4.1.0 and in the Russian Federal Medical and Biological Agency database of population frequencies of genetic variants for the Russian Federation population (version 1.1.2, database version 59.1, https//gdbpop.nir.cspfmba.ru). This missense variant is located in the gene where missense variants often cause the disease.

The couple underwent detailed medical genetic counseling, including the issues of planned molecular genetic testing system and ART (assisted reproductive technologies) counseling. Voluntary informed consent was obtained from all persons involved in the study for all procedures

The biological material for the PGT-M setup was venous blood samples from spouses and parents of the husband, as well as from a voluntary unrelated donor (for DNA testing and single lymphocytes) collected into EDTA-containing vacutainer tubes. For postnatal diagnosis, the newborn’s dried blood spots on a filter blank were used. DNA isolation was carried out using the DNA-sorb-B kit (Amplisens, Russia) from 100 μl blood. DNA from a dried blood spot sample was isolated using the PREP-MB-DBS DWP kit (DNA-Technology, Russia) at the Auto-Pure 96 robotic station (Hangzhou Allsheng Instruments Co., Ltd, China). Samples of single lymphocytes were used as biological material to validate the system. For this purpose, a suspension of mononuclear cells was isolated from blood by centrifugation in a density gradient of ficoll solution. Single lymphocytes were then taken with a glass biopsy micropipette under a microscope using a polyvinylpyrrolidone solution and each was placed into an individual microtube with a lysing solution. The lysing solution for single lymphocytes and embryo trophectoderm samples contained proteinase K, triton X-100, and twin 20 (Verlinsky, Kuliev, 2004).

For PGT-M, a testing system has been developed that includes analysis of the pathogenic variant D336G of the FGFR2 gene, as well as polymorphic STR markers (short tandem repeats) linked to the FGFR2 gene. STRs have been selected within 1 million bp from the pathogenic variant with heterozygosity ranging from 0.8 to 0.9 for dinucleotide repeats, and no less than 0.7 for tri-tetranucleotide repeats. At the PGT-M setup stage, PCR reactions and optimization (gradient PCR if necessary) were performed for all fragments for test DNA, followed by analysis of family samples to identify informative STRs. The developed system was validated on single lymphocytes (N = 8).

Standard PCR (2nd PCR) in a volume of 20 μl with fluorescently labeled oligonucleotide primers was used to test DNA samples from family members. Nested PCR was used during single-cell testing, both during PGT-M and single lymphocyte validation. The first round of PCR (1st PCR) was multiplexed in a volume of 50 μl. 1 μl of the 1st PCR product was used as a template for the 2nd PCR. Negative and positive controls were used in all amplification series. The overall scheme of molecular genetic testing, as well as conditions and amplification programs were carried out according to the recommendations of Y. Verlinsky and A. Kuliev (Verlinsky, Kuliev, 2005).

The 2nd PCR products were preliminarily examined in a 7 % polyacrylamide gel to assess the loading of samples on capillary electrophoresis and the quality of negative controls. Fragment analysis of STR markers was performed by capillary electrophoresis on a Nanophore 05 genetic analyzer (Institute of Analytical Instrumentation, Russia). The GeneMarker software (SoftGenetics, USA) was used to evaluate the fragment analysis results. The study of the pathogenic variant involved restriction analysis with HspAI and detection in a 7 % polyacrylamide gel. GelRed dye (Biotinum, USA) was used to color gels.

The couple underwent standard pre-treatment assessment according to national guidelines to plan IVF (Assisted Reproductive Technology…, 2019). Controlled ovarian hyperstimulation was performed using standard short protocol with recombinant FSH (follicle stimulating hormone) and antagonists. The dose of FSH was selected individually. Ovulation was triggered 0.2 mg of Decapeptil (Ferring GmbH, Germany) when at least three follicles reached 17 mm in size according to ultrasound monitoring. Transvaginal follicle puncture was performed under ultrasound control 36 hours later. WHO standard criteria were used for ejaculate analysis (WHO Laboratory Manual…, 2010).

ICSI (intracytoplasmic sperm injection) was used as a fertilization technique. Embryological procedures and embryo biopsy were carried out taking into account national and international guidelines (ESHRE PGT Consortium and SIGEmbryology Biopsy Working Group et al., 2020; Evaluation of Oocytes…, 2021). A single-step medium SAGE 1-Step™ (ORIGIO, Denmark) was used for embryo culture. Biopsy of good and excellent quality embryos was performed on day 6 using a flip method. The obtained trophectoderm fragments, after washing in phosphate buffer (PBS), were transferred into microtubes containing 5 μl of lysing buffer and frozen at –20 °C. Immediately after the biopsy, blastocysts were vitrified using Kitazato media and carriers (Kitazato Corporation, Dibimed-Biomedical Supply, Spain).

PGT-M was performed using the nested PCR method, according to the scheme developed at the preliminary stage, taking into account the aforementioned conditions and international recommendations (ESHRE PGT-M Working Group et al., 2020).

The preparation for the transfer of the thawed embryo was carried out in a natural cycle, taking into account the results of PGT-M. Kitazato thawing media (Kitazato Corporation, Dibimed-Biomedical Supply, Spain) and a transfer catheter Guardia™ Access ETEmbryo Transfer Catheter (COOK Medical, USA) were used.

Pregnancy diagnosis was performed by standard analysis of human chorionic gonadotropin (HCG) on the 14th day after the embryo transfer, followed by ultrasound examination of the gestation at 7 weeks. No invasive prenatal diagnosis was made.

Postnatal diagnosis was performed using samples of dried newborn bloodstains. Molecular genetic testing of the newborn included all the loci tested during PGT-M.

The study was carried out using the resources of the Biobank of the Population of Northern Eurasia biocollection at the Research Institute of Medical Genetics of the Tomsk National Research Medical Center and the equipment of the Medical Genomics Center for Collective Use at the Tomsk National Research Medical Center.

## Results

During the PGT-M setup, a test system was developed for a family with a high genetic risk (50 %) of Crouzon syndrome, including the analysis of the pathogenic variant D336G and 12 polymorphic STR markers. The presence of the pathogenic variant was confirmed by restriction analysis in the husband and his father, both for the preliminary examination of the family and for the PGT-M (see the Figure).

**Fig. 1.**
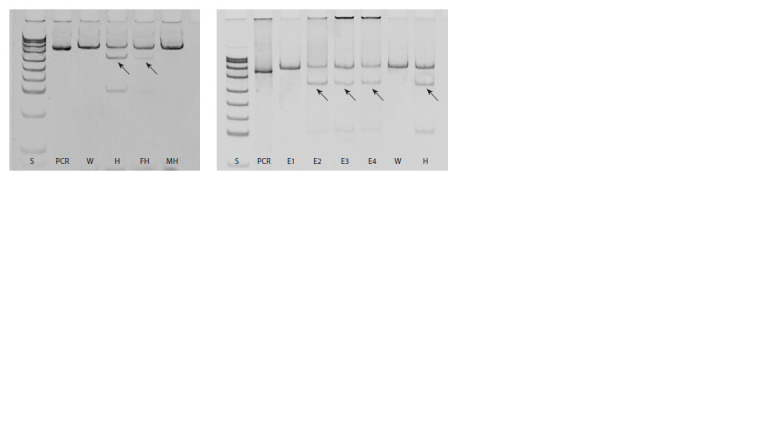
The result of testing the pathogenic variant D336G in the FGFR2 gene by restriction analysis with gel detection (inverted image) in family samples from PGT-M setup and embryo samples during PGT-M. W – wife/woman; H – husband/man; FH – husband’s father; MH – husband’s mother; E1–E4 – the embryo samples; PCR – PCR product without endonuclease digestion; S – the pUC19/MspI size standard.

We have recorded a difference in the intensity of restriction fragments between the father and the husband. In the husband’s father, the fragment corresponding to the allele with the pathogenic variant looked significantly paler than normal. This was consistent with the results of molecular genetic examination provided by the family for PGT-M planning, which also noted mosaicism in the husband’s father.

The informative value of STR markers for PGT-M was established through the family analysis based on testing samples from the husband’s parents. Table 1 shows the polymorphic STR markers we selected and studied in the family samples

**Table 1.**
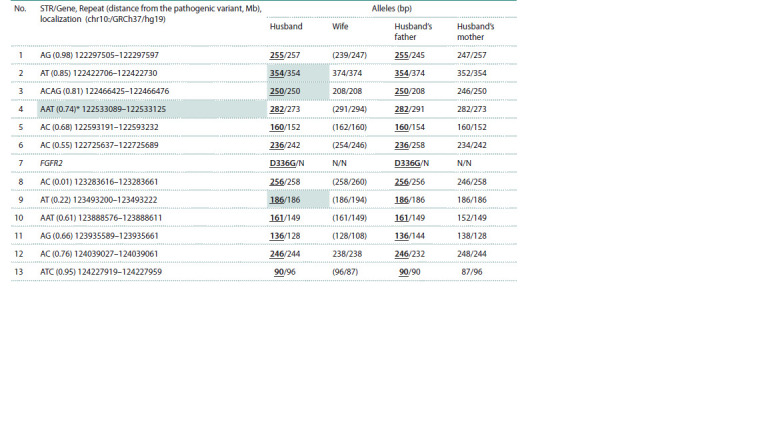
Results of the preliminary stage of PGT-M for a family with Crouzon syndrome Notе. Alleles linked to the pathogenic variant are indicated in bold and underlined; uninformative STRs are marked in gray; alleles with coupling that were not established during the preliminary stage are marked in parentheses. * A locus that has not been validated on single cells is marked.

Nine of the twelve STRs tested were informative regarding the husband’s chromosome carrying the pathogenic variant. The husband’s father did not have any additional alleles across the entire spectrum of highly polymorphic microsatellite markers studied, which excluded the assumption of chimerism.

Next, the testing system was validated using samples of single lymphocytes and products obtained from wholegenome amplification of embryos. Acceptable amplification in terms of fragment intensity, absence of non-specific fragments and peak shape was confirmed for all STRs except for one. One STR located at a distance of 0.74 million bp was excluded from the system due to lack of amplification on single cells.During the IVF treatment cycle, a starting dose of gonadotropins 200 IU FSH + 75 IU LH (luteinizing hormone) was used. The total gonadotropin dose was 1,950 IU FSH + 600 IU LH. No complications occurred during stimulation. 21 oocytes were retrieved from 23 follicles, 15 of them being mature. Fertilization was performed by ICSI in order to reduce contamination of parental DNA during embryo testing. The husband’s ejaculate was teratozoospermic; the sperm concentration was 167 million/ ml, the per-centage of progressively mobile was 49 %. The next day after fertilization, 12 zygotes with two pronuclei were formed. On day 3, 11 embryos were developing. Four embryos have reached the blastocyst stage, and all of them have been successfully biopsied for genetic testing.Trophectoderm fragments from four embryos were tested for the familial pathogenic variant responsible for Crouzon syndrome and the linked STR markers selected at the preliminary stage. The preimplantation study included only informative STRs validated on single cells

The results of preimplantation analysis of pathogenic variant of the FGFR2 gene are shown in the Figure. During PGT-M, the paternal pathogenic variant D336G of the FGFR2 gene was found in a heterozygous state in three embryo samples. A normal FGFR2 allele was detected in the sample of the first embryo. The genotype of embryo 1 can be interpreted as homozygous for the normal allele. However, due to the risk of allele dropout (ADO) in PGT- M, the genotype was interpreted only in conjunction with STR results. The full results of preimplantation testing are presented in Table 2.

All presumably homozygous profiles in the embryo samples (Table 2) are indicated as a single allele, as a precautionary measure in interpretation, due to the possible phenomenon of ADO. The results of STR testing showed that the samples from three embryos carrying the pathogenic variant contained paternal alleles linked to the pathogenic variant of the gene. A normal paternal haplotype was detected in embryo 1, along with one of the maternal haplotype, which confirmed the normal homozygous status of the embryo in relation to the pathogenic variant.

**Table 2.**
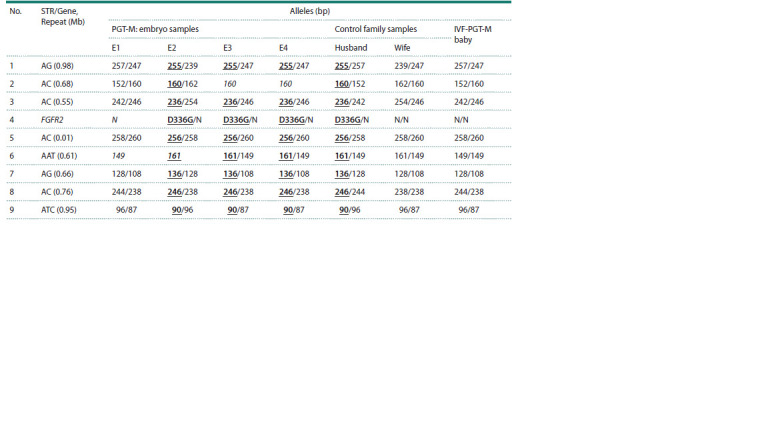
PGT-M results and confirmatory diagnostic results for a family with Crouzon syndrome Notе. Alleles linked to a pathogenic variant are shown in bold and underlined; loci in italics can be interpreted individually in embryo samples as homozygous or one allele due to the ADO of the second allele.

Genetic testing for aneuploidy was not carried out, based on the couple’s decision following medical genetic counseling.The transfer of thawed embryo number 1 into the uterine cavity was performed based on the preimplantation testing results. Cryotransfer was performed in a naturally modified ovulation trigger cycle (HCG 6,500 IU).

The result was a singleton pregnancy, confirmed by ultrasound. The pregnancy progressed without complications. Standard prenatal screening did not detect any fetal abnormalities. Invasive prenatal diagnosis, recommended by genetic counseling to check the genotype of the fetus, was not performed due to the patient’s concerns about potential complications. The pregnancy was terminated via cesarean section at 39 weeks of gestation. In May 2024, a healthy baby girl weighting 3,480 g was born. Her Apgar score was 7/8 according to the discharge records.

Confirmatory genetic diagnosis was performed using dried newborn blood sample for the familial variant of Crouzon syndrome. The testing was carried out on all loci included in the PGT-M (Table 2). As a result, the child had a genotype homozygous for the normal allele of the FGFR2 gene, confirmed by the paternal haplotype. The postnatal confirmatory diagnosis results completely matched the results of embryo 1 testing during PGT-M.

## Discussion

Our article provides a detailed description of a clinical case of IVF with PGT-M to prevent the birth of a child with Crouzon syndrome in a family where the husband and his father suffered from this disease. The familial pathogenic variant of the FGFR2 gene in the husband’s father was present in a mosaic form and was not a consequence of chimerism, as proven by our data from microsatellite analysis. This most likely caused a milder clinical manifestation of the disease. For embryos, we recorded the standard heterozygous (nonmosaic) pathogenic variant, as well as for the husband (see the Figure).

A case of generative and somatic mosaicism for the same pathogenic variant c.1007A>G(p.Asp336Gly), as in our clinical case, is described in the work of A. Goriely et al. (2010). In a mother without Crouzon syndrome, a heterozygous mosaic variant c.1007A>G(p.Asp336Gly) was detected in approximately 25–30 % of blood and saliva cells. In a child with Crouzon syndrome, this pathogenic variant was present, as it was in our case, in the standard heterozygous form (Goriely et al., 2010).Cases of transmission of an autosomal dominant disease from a parent who is a mosaic carrier of a pathogenic variant have also been found for other conditions, in particular, for autosomal dominant polycystic kidney disease (Hopp et al., 2020). The prevalence of this phenomenon is currently unclear for various hereditary diseases

During the IVF program, four blastocysts were obtained, all of which were successfully biopsied and tested for Crouzon syndrome. We used a strategy based on analyzing the pathogenic variant and linked informative STR markers that were selected during the PGT-M setup. We used nested PCR but not genome-wide amplification, because the combination of PGT-M and aneuploidy analysis was not performed for this clinical case. Our approach allowed us to draw definitive conclusions regarding the inheritance of the chromosome responsible for the familial variant of Crouzon syndrome in each embryo. The dominant nature of inheritance of the disease determines a 50 % risk for embryos. In our case, 3 out of 4 (75 %) embryos had the pathogenic variant. One embryo had a normal status, and its transfer led to the achievement of pregnancy and birth of a healthy child.

There is little data on PGT-M for Crouzon syndrome in scientific publications. In 2002, a group from the UK published the results, reporting the first successful PGT for this condition (Abou-Sleiman et al., 2002; Harper et al., 2002). Both articles describe the same clinical case. One article presents the test results in more detail (Abou-Sleiman et al., 2002), while the second one focuses on the outcomes (Harper et al., 2002). A married couple, in which a woman had Crouzon syndrome, underwent pre-implantation testing during two IVF cycles. They had a history of the birth of a girl with Crouzon syndrome, who later died during corrective surgery. The preimplantation testing system included the analysis of the pathogenic variant using the SSCP (single strand conformational polymorphism) method. Two intragenic SNPs were tested during preparation for the diagnosis, but they were uninformative for the spouses (Abou-Sleiman et al., 2002). In addition to the FGFR2 gene fragment, unlinked D21S11 locus was included in the preimplantation analysis to control amplification. In just two cycles, 23 day 3 embryos were analyzed. The strategy of taking two blastomeres during biopsy was used. Eight embryos were diagnosed as normal, nine as abnormal and six were inconclusive. Five embryos were transferred during two embryo transfer procedures. The second cycle of PGT resulted in a twin pregnancy, but only one embryo had a heartbeat. Prenatal diagnosis was not performed at the request of the married couple and confirmatory diagnosis was done postnatally. A healthy baby boy was born (Harper et al., 2002).We have not found any other descriptions of PGT-M for Crouzon syndrome. During our work, we did not encounter any specific difficulties with PGT-M related to the disease, gene, or specific pathogenic variant. Due to the complex molecular mechanisms involved in craniosynostosis development, this group may have attracted less attention from specialists as a method for preventing the disease until now. Our experience shows that PGT-M can successfully be applied in cases where there is a clear monogenic inheritance.

In comparison with the above-described clinical case from the literature, in our case, pregnancy was achieved in the first treatment cycle of IVF with PGT. The differences can be noted in a number of aspects of the entire process related to technology changes in this area. We had fewer embryos for preimplantation testing. In general, when testing trophectoderm samples from 5 or 6-day embryos, as it was in our study, there are fewer samples for PGT-M compared to testing blastomeres on day 3, because of natural selection of embryos from day 3 to 5.For the genetic analysis we used a more detailed testing system, which included a clear identification of the pathogenic variant by restriction analysis, and at least two informative flanking STRs for the family. To analyze the pathogenic variant properly, a restriction analysis was used based on the natural site of the restriction endonuclease in the case of the pathogenic variant. This approach proved to be more informative than the SSCP method. Currently, a wide range of innovative genome-wide methods have been proposed in the field of PGT-M (De Rycke, Berckmoes, 2020). At the same time, it is important to consider the potential benefits and drawbacks of these methods for each specific case. The STRs included in our study allowed us to identify all the difficulties of amplification and interpretation of PGT results, including ADO, contamination, and recombination. Even in the cases where amplification of the pathogenic allele fails, haplotypes can allow us to clearly identify embryos carrying a chromosome with the paternal pathogenic variant. According to modern standards, targeted preimplantation analysis without linked informative markers is not recommended (ESHRE PGT-M Working Group et al., 2020).

In the clinical case presented by us, PGT for aneuploidy was not performed. The spouses were of a relatively young age; there were no other indications for this procedure.

## Conclusion

Our study presents a clinical case of IVF with PGT-M for a married couple at high risk for Crouzon syndrome, with a successful outcome confirmed by postnatal diagnosis. In comparison with the only case presented in the literature, our results reflect a more modern and, in some aspects, a more reliable approach to IVF with PGT-M for Crouzon syndrome. In the family studied, the pathogenic variant of the FGFR2 gene was present in a mosaic form in the husband’s father, while in the husband and, consequently, in embryos it had the standard heterozygous state. Microsatellite analysis used in our work excluded chimerism in the husband’s father. The issues of transmission of dominant diseases in the cases of parental mosaicism require further research. The clinical case presented by us demonstrates an effective example of the use of IVF with PGT-M to prevent the birth of sick children in families with hereditary craniosynostosis.

## Conflict of interest

The authors declare no conflict of interest.
